# Neurodegenerative disease biomarkers Aβ_1–40_, Aβ_1–42_, tau, and p‐tau_181_ in the vervet monkey cerebrospinal fluid: Relation to normal aging, genetic influences, and cerebral amyloid angiopathy

**DOI:** 10.1002/brb3.903

**Published:** 2018-01-13

**Authors:** Jason A. Chen, Scott C. Fears, Anna J. Jasinska, Alden Huang, Noor B. Al‐Sharif, Kevin E. Scheibel, Thomas D. Dyer, Anne M. Fagan, John Blangero, Roger Woods, Matthew J. Jorgensen, Jay R. Kaplan, Nelson B. Freimer, Giovanni Coppola

**Affiliations:** ^1^ Department of Psychiatry The Jane and Terry Semel Institute for Neuroscience and Human Behavior David Geffen School of Medicine University of California Los Angeles CA USA; ^2^ Interdepartmental Program in Bioinformatics University of California Los Angeles CA USA; ^3^ Verge Genomics San Francisco CA USA; ^4^ Department of Psychiatry Greater Los Angeles Veterans Administration Los Angeles CA USA; ^5^ Institute of Bioorganic Chemistry Polish Academy of Sciences Poznan Poland; ^6^ South Texas Diabetes and Obesity Institute University of Texas Rio Grande Valley School of Medicine Brownsville TX USA; ^7^ Department of Neurology Washington University in St. Louis St. Louis MO USA; ^8^ Department of Neurology David Geffen School of Medicine at UCLA University of California Los Angeles CA USA; ^9^ Department of Pathology Section on Comparative Medicine Wake Forest School of Medicine Winston‐Salem NC USA

**Keywords:** Alzheimer's disease, amyloid beta, cerebral amyloid angiopathy, cerebrospinal fluid, tau, vervet

## Abstract

**Background:**

The Caribbean vervet monkey (*Chlorocebus aethiops sabaeus*) is a potentially valuable animal model of neurodegenerative disease. However, the trajectory of aging in vervets and its relationship to human disease is incompletely understood.

**Methods:**

To characterize biomarkers associated with neurodegeneration, we measured cerebrospinal fluid (CSF) concentrations of Aβ_1–40_, Aβ_1–42_, total tau, and p‐tau_181_ in 329 members of a multigenerational pedigree. Linkage and genome‐wide association were used to elucidate a genetic contribution to these traits.

**Results:**

Aβ_1–40_ concentrations were significantly correlated with age, brain total surface area, and gray matter thickness. Levels of p‐tau_181_ were associated with cerebral volume and brain total surface area. Among the measured analytes, only CSF Aβ_1–40_ was heritable. No significant linkage (LOD > 3.3) was found, though suggestive linkage was highlighted on chromosomes 4 and 12. Genome‐wide association identified a suggestive locus near the chromosome 4 linkage peak.

**Conclusions:**

Overall, these results support the vervet as a non‐human primate model of amyloid‐related neurodegeneration, such as Alzheimer's disease and cerebral amyloid angiopathy, and highlight Aβ_1–40_ and p‐tau_181_ as potentially valuable biomarkers of these processes.

## 1 INTRODUCTION

Nonhuman primates, unlike most other model organisms (e.g. mice and rats), are known to develop age‐related amyloid pathology similar to that of Alzheimer's disease (AD) in humans (Kalinin et al., 2013; Ndung'u et al., 2012; Podlisny, Tolan, & Selkoe, 1991; Rosen et al., 2008; Toledano, Álvarez, López‐Rodríguez, Toledano‐Díaz, & Fernández‐Verdecia, 2014), and have complex behavioral phenotypes closer to that in humans than other species could achieve. AD‐related genes, such as those encoding β‐amyloid (Aβ, *APP*) and tau (*MAPT*), are also highly similar (Holzer, Craxton, Jakes, Arendt, & Goedert, 2004; Podlisny et al., 1991). Tau pathology, though reported in great apes, baboons, and lesser primates, appears far less abundant than in human disease, and distribution of amyloid pathology differs from that in human AD (Heuer, Rosen, Cintron, & Walker, 2012; Toledano et al., 2014). Correlation of amyloid pathology with cognition and atrophy has been observed, but to a lesser degree compared with humans (Heuer et al., 2012; Toledano et al., 2014).

The Caribbean vervet monkey (*Chlorocebus aethiops sabaeus*, also known as the African green monkey), an Old World monkey, is well‐suited as a model of aging and neurodegenerative disease; compared to rhesus macaques, vervets are more available and carry fewer pathogens. Vervets display AD‐like pathology, including accumulation of Aβ‐containing cerebral plaques associated with reactive astrocytosis, dystrophic neurites, and vascular Aβ immunoreactivity resembling cerebral amyloid angiopathy (CAA), but unlike humans have relatively preserved cognition that does not appear correlated with plaque burden and demonstrate scant phosphorylated tau aggregates (Heuer et al., 2012; Kalinin et al., 2013; Lemere et al., 2004; Toledano et al., 2014). The high incidence of Aβ immunoreactive plaques in vervets older than 20 years of age (Kalinin et al., 2013) suggests that they occur as part of the normal aging of the vervet.

In humans, cerebrospinal fluid (CSF) harbors useful biomarkers of neurodegenerative disease. CSF levels of Aβ and tau change with AD. Aβ exists in several isoforms, predominantly composed of residues 1–40 (Aβ_1–40_) and residues 1–42 (Aβ_1–42_). Aβ_1–40_ is the most abundant isoform, but CSF concentration differences of Aβ_1–40_ are generally subtle in AD (Fagan et al., 2007, 2014). Indeed, the shorter Aβ_1–40_ isoform is more predominant in CAA, and a decrease in CSF Aβ_1–40_ is associated with CAA (Verbeek et al., 2009). Aβ_1–42_ is less abundant, but constitutes the major isoform present in neuritic plaques in humans and in vervets and is more fibrillogenic. CSF Aβ_1–42_ has been consistently shown to be decreased in AD (Andreasen et al., 1999; Fagan et al., 2014; Motter et al., 1995; Sunderland et al., 2003; Tapiola et al., 2009). Some authors have reported that the Aβ_1–42_/Aβ_1–40_ ratio is a more accurate biomarker of AD than Aβ_1–42_ alone (Dumurgier et al., 2015; Hansson et al., 2007; Lewczuk, Lelental, Spitzer, Maler, & Kornhuber, 2015; Wiltfang et al., 2007). CSF tau and phosphorylated tau, particularly at Threonine 181, are increased in AD patients (Andreasen et al., 1999; Fagan et al., 2014; Sunderland et al., 2003; Tapiola et al., 2009; Vandermeeren et al., 1993), however the mechanism of tau egress into the CSF and the relation to tau pathology is unclear. These CSF biomarkers have also been shown to change longitudinally in patients with familial forms of AD and predict clinical disease progression (Bateman et al., 2012; Fagan et al., 2007; Hansson et al., 2006). Collection of CSF enables the study of amyloid pathology across a large sample of vervets and is a first step toward understanding the translatability of CSF biomarkers between human and vervet.

The Vervet Research Colony (VRC) was established from vervets captured in St. Kitts and Nevis, and maintained as a single extended pedigree (Jasinska et al., 2012), thereby providing a large sample ideal for mapping quantitative traits and study of complex neurobiologic traits. Additionally, the wide distribution of subjects with ages across the normal vervet lifespan enables the study of age‐related disease; vervets typically live to 11–13 years in the wild, but up to 25 years in captivity (Magden, Mansfield, Simmons, & Abee, 2015); the oldest animal in the VRC was a female that recently died at 29.1 years of age. Previous work in the VRC reported on the acquisition and analysis of neuroanatomic phenotypes using high‐resolution structural MRI in a large number of colony members (Fears et al., 2009, 2011). In the current study, we extend the neurobiologic characterization of the VRC through the acquisition and analysis of CSF biomarkers in the context of the rich genetic and phenotypic information available through the VRC.

## 2 METHODS AND MATERIALS

### 2.1 Vervet pedigree

This investigation was conducted as approved by the Animal Research Committee in the Office for Protection of Research Subjects at UCLA and the Institutional Animal Care and Use Committees at the Sepulveda Veterans Administration Medical Center and the Wake Forest School of Medicine. The vervet subjects analyzed in this study are part of a pedigree that has included more than 1,000 animals since its founding. The VRC was established during the 1970s and 1980s from 58 founder animals that were captured from a wild population in the islands of St. Kitts and Nevis. Vervet monkeys originally arrived in the Caribbean Islands in the 1600s on trading ships from Africa (McGuire, 1974). Since the founding of the colony, breeding has been managed to provide a species‐typical social environment for developing offspring and to promote genetic diversity, while preserving each of the original matrilines to simulate the social structure of wild‐living troops. Female offspring remain in the breeding groups, and males are removed to separate housing at 4 years of age as previously described (Fairbanks et al., 2004). Adult males born in different social groups are introduced into the breeding groups at 3–4 year intervals; because no new animals have been imported for several decades, extensive inbreeding occurs. The average inbreeding coefficient (probability that a subject receives two alleles identical‐by‐descent) in the colony was recently characterized at 0.006, with animals with non‐zero inbreeding coefficient ranging from 0.004 to 0.25 (mean 0.05; Freimer et al., 2007). The high level of inbreeding facilitates genetic mapping in our study population, improving the effective sample size for association and providing a high informative pedigree for linkage. The genetic architecture and genetic control of traits may not reflect outbred populations.

Vervets were fed commercial laboratory chow ad lib, supplemented by fresh fruits and produce. Specifically, the vervet chow has primarily been LabDiet 5038 (“Monkey Diet”), with several exceptions. During 2004 to 2008, animals were fed LabDiet 5052 (“Fiber‐Balanced Monkey Diet”) as previously described (Fairbanks, Blau, & Jorgensen, 2010). For 6 months in mid 2009, animals were fed LabDiet 5L0P (“Typical American Primate Diet”) as previously described (Voruganti et al., 2013).

### 2.2 CSF biomarker measurements

CSF was collected from the cisterna magna of each animal under a protocol approved by the Animal Research Committee at the University of California, Los Angeles, as previously described (Freimer et al., 2007). CSF protein concentrations were measured at the Knight Alzheimer's Disease Research Center (ADRC) Biomarker Core Lab at Washington University in St. Louis as previously described (Shaw et al., 2011). Concentrations of CSF Aβ_1–40_ were measured with the INNOTEST β‐AMYLOID_(1–40)_ solid‐phase enzyme immunoassay (Innogenetics N.V., Ghent, Belgium). Briefly, the amyloid peptides are first captured by the 2G3 monoclonal antibody, and quantified with a biotinylated monoclonal antibody, 3D6. The protein is then detected using a peroxidase‐labeled streptavidin readout. Concentrations of CSF Aβ_1–42_, tau, and phosphorylated tau (p‐tau_181_) were measured simultaneously with the INNO‐BIA AlzBio3 fluorimetric immunoassay (Innogenetics N.V., Gent, Belgium). Briefly, the proteins are first captured on beads by specific monoclonal antibodies – 4D7A3 for Aβ_1–42_, AT120 for tau, and AT270 for p‐tau_181_. Additional biotinylated antibodies are then used to quantify the proteins – 3D6 for Aβ_1–42_, and HT7 for tau and p‐tau_181_. The proteins are then detected using a phycoerythrin‐labeled streptavidin readout. Calibration of the standard curve was performed as described in the manufacturer's directions using six accompanying standards provided in the kit. All assays were run in duplicate. Measurements with coefficient of variation (CV) >25% or bead count <30 were excluded in quality control.

### 2.3 Neuroimaging analysis

Neuroimaging data were acquired in 2007; details of the image acquisition and pre‐processing protocol have been described previously (Fears et al., 2009). To generate high signal‐to‐noise images, nine separate structural scans were acquired from 357 animals (256 females and 101 males) using an 8‐channel high‐resolution knee array coil as a receiver in a 1.5 Tesla Siemens (Erlanger) Symphony unit. The images were acquired as axial T1‐weighted volumes with a 3D magnetization prepared rapid acquisition gradient echo (MPRAGE); TR = 1,900 msec, TE = 4.38 msec, TI = 1,100 msec, flip angle = 15 degrees, and voxel resolution = 0.5 mm in all three planes. The nine separate images were aligned to each other in pair‐wise rigid body registrations and averaged together prior to segmentation. The age at CSF biomarker collection and measurement (performed in 2012) was approximately 5 years following the age at MRI measurement (performed in 2007); analyses were performed with each animal from the CSF analysis paired with their own earlier scan data.

Details of the six brain phenotypes investigated in the initial association analysis have been described previously (Fears et al., 2009). For the secondary analysis of regional cortical phenotypes, the images were segmented to generate cortical thickness and surface area measures using a combination of manual and automated methods. Thirty images were manually segmented into 29 regions based on each subject's anatomical details. The 30 manually segmented images were used as input for the Freesurfer (RRID:SCR_001847) program ‘mri_ca_train’ to generate a probabilistic anatomical map (https://surfer.nmr.mgh.harvard.edu/fswiki/mri_ca_train) that was then used to segment the entire dataset. The Freesurfer segmented images were then inspected and manually adjusted to correct gross segmentation errors.

### 2.4 Statistical analysis

Brain‐CSF associations were tested using linear regression as implemented with the Sequential Oligogenic Linkage Analysis Routines (SOLAR) software package (Almasy & Blangero, 1998; RRID:SCR_000850). SOLAR uses the pedigree structure to adjust for the dependency structure among the subjects that would otherwise violate assumptions for standard linear regression methods. For the neuroimaging measures, age and sex were controlled as covariates in a linear regression model; the residuals were then associated with CSF traits. The procedure of Benjamini and Hochberg was used to correct for multiple testing to identify significant associations in the initial set of six neuroimaging traits with the four CSF traits (24 tests), using a false discovery rate (FDR) threshold of 0.05. In the secondary analysis of regional cortical measures of thickness and surface area with Aβ_1–40_ and p‐tau_181_ traits, the procedure of Benjamini and Hochberg was similarly used, applied to each of the two sets of association tests independently (29 tests per set).

### 2.5 Heritability calculations and linkage analysis

A whole‐genome sequencing strategy was used to identify and type vervet polymorphisms, as previously described (Huang et al., 2015). The vervet pedigree was visualized using the “kinship2” package. Heritability analysis was performed using SOLAR. The variance component models of SOLAR assume normality in the trait distributions; therefore, in order to force the distribution of CSF biomarker phenotypes to approximately normal and correct for skew and kurtosis, the inverse normal transformation was used. For each phenotype, the correlation to age and sex covariates was evaluated. Residuals after regression for significant covariates were used in downstream analyses. Quantitative polygenic screening was performed as previously described using the “polygenic” function of SOLAR, yielding an estimate of the narrow‐sense polygenic heritability (*h* ^2^, a measure of the fraction of phenotypic variance due to additive genetic factors); a *p*‐value for this estimate; and the proportion of variance attributable to covariates. Genetic mapping was performed using multipoint linkage analysis implemented in SOLAR. First, the multipoint identity‐by‐descent (IBD) relationships were computed for the vervet pedigree as previously described (Huang et al., 2015). A multipoint linkage scan was then run with an interval of 5 centimorgans. LOD scores were considered suggestive at a threshold of 1.9, and significant at a threshold of 3.3 as previously proposed (Lander & Kruglyak, 1995). Zero‐in scans at finer scales were performed at peaks with LOD scores >2. The confidence region around a peak was defined as the region spanned by 1 LOD intervals to either side of the peak.

### 2.6 Association testing

The EMMAX software was used to map genetic associations with CSF biomarker phenotypes in the vervet (Kang et al., 2010; RRID:SCR_008217). EMMAX uses a variance component model to account for high relatedness among the vervet subjects (estimated from pairwise kinship calculated by identity‐by‐state). A linear mixed model was then fit to the data and used to calculate the association. We used standard genome‐wide significant thresholds from humans (significant at *p* < 5 × 10^−8^, suggestive at *p* < 5 × 10^−6^; Risch & Merikangas, 1996), which should approximate appropriate values in vervet given the relatively recent divergence between apes and Old World Monkeys (approximately 25 million years), and the similarity in genome size between human and vervet (Warren et al., 2015).

## 3 RESULTS

### 3.1 Vervet subject characteristics

Animals were selected from a complex, nine‐generation‐deep vervet pedigree with a high degree of consanguineous mating. Following quality control procedures (see Methods), CSF concentrations of Aβ_1–40_, Aβ_1–42_, tau, and p‐tau_181_ (tau phosphorylated at Thr181) were obtained in a total of 329 animals (Table 1). Unexpectedly, for each demographic category, mean total tau concentrations were lower than those of p‐tau_181_. The distributions of CSF concentrations of Aβ_1–40_, Aβ_1–42_, tau, and p‐tau_181_ were skewed (Table 1, Figure S1). Levels of Aβ_1–40_ were inversely correlated with age (*r* = −.17, *p* = .00094), while levels of Aβ_1–42_, tau, and p‐tau_181_ were not (*r* = −.039, .0086, .07, and *p* = .47, .87, and .19, respectively; Figure S2). The correlation between age and CSF Aβ_1–40_ was consistent when excluding animals >10 years of age (*r* = −.13, *p* = .093).

**Table 1:** Characteristics of vervet subjects used in the analysis, at the time of CSF collection

	*N* (%)	Ab40 mean (*SD*), pg/ml	Ab42 mean (SD), pg/ml	Tau mean (*SD*), pg/ml	pTau mean (*SD*), pg/ml
All	329 (100)	10267 (4250)	506 (166)	23 (6)	30 (11)
0–5 years old	87 (26)	11047 (3880)	505 (181)	23 (5)	30 (10)
5–10 years old	99 (30)	10282 (3819)	503 (169)	22 (6)	29 (12)
10–15 years old	78 (24)	10094 (4509)	499 (162)	23 (8)	28 (11)
15–20 years old	45 (14)	10289 (5332)	499 (168)	23 (8)	33 (11)
20+ years old	20 (6)	7416 (2939)	556 (92)	22 (4)	36 (14)
Male	55 (17)	11151 (4371)	529 (154)	21 (2)	33 (11)
Female	274 (83)	10088 (4211)	501 (168)	23 (7)	29 (11)

### 3.2 Species differences and polymorphisms in the vervet β‐amyloid and tau proteins

The antibody‐based CSF assays were designed and validated for humans; therefore, we evaluated whether species‐specific differences or polymorphisms in the vervet orthologs of *APP* (encoding the amyloid precursor protein) and *MAPT* (encoding tau) could affect the performance of the antibodies.

Species‐specific differences between vervet and human in the antibody epitope regions were first identified. Across the Aβ peptide the human and vervet proteins were identical, including the 2G3, 4D7A3, and 3D6 epitope regions (Figure S3). Differences were found between the human tau protein and the vervet ortholog, including a single amino acid substitution (threonine to alanine) in the AT120 epitope region, which could affect the results for total tau concentration.

Coding polymorphisms across vervet subjects were then identified to determine whether inter‐individual differences in the polypeptide sequence of Aβ or tau could confound their quantification. In our cohort, polymorphisms were identified in APP (Ala15Ser) and MAPT (Gly148Arg and Leu213Pro). None overlapped with the antibody epitope regions; furthermore, no associations between these polymorphisms and quantified CSF concentrations were observed (Figure S4).

### 3.3 Relation between CSF biomarker level and brain structure

**Figure 1:**
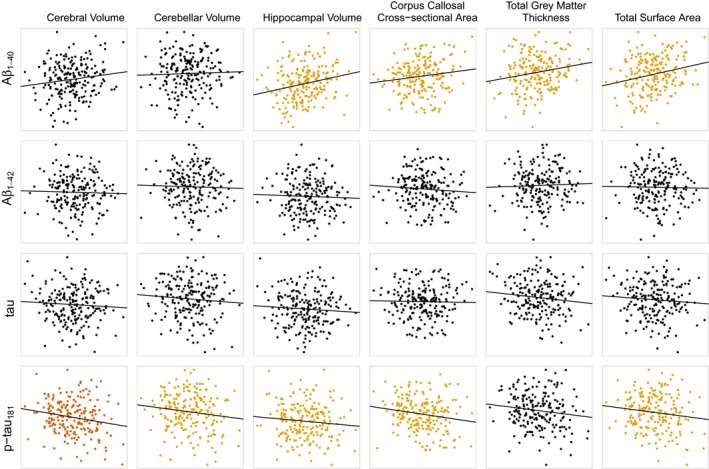
Associations between global measures of brain size and CSF biomarkers (Aβ_1–40_, Aβ_1–42_, tau, and p‐tau_181_) in 209 vervets with both CSF collection and MRI scan. Colored points denote the strength of association – non‐significant association (*p* > .05, black), suggestive association (*p* < .05, yellow), and significant association (Benjamini‐Hochberg corrected *p* < .05, red)

Brain imaging phenotypes and genetic data had been collected as part of a previous study (Fears et al., 2009). In total, both CSF biomarker measurements and neuroimaging data were measured in 209 animals. In general, this subgroup had fewer animals of very young age, due to the time interval between studies (Figure S5). To determine the relationship between the CSF concentrations of Aβ_1–40_, Aβ_1–42_, tau, and p‐tau_181_ with neuroimaging phenotypes, we initially calculated the correlations of CSF biomarker concentrations with six global measures (cerebral volume, cerebellar volume, hippocampal volume, cross‐sectional area of the corpus callosum, total gray matter thickness, and total surface area; see Table 2 and Figure 1), after correction for age and sex. At a nominal significance threshold (*p* = .05), Aβ_1–40_ was correlated with corpus callosum cross‐sectional area (*p* = .024), hippocampal volume (*p* = .013), total gray matter thickness (*p* = .007), and total surface area (*p* = .006). The correlation with total gray matter thickness and total surface area was significant (controlling FDR at 0.05) after applying the Benjamini‐Hochberg correction for multiple comparisons. The direction of each regression coefficient was positive; higher concentrations of Aβ_1–40_ were associated with larger brain size. Likewise, p‐tau_181_ concentrations were correlated with cerebral volume (*p* = .001), cerebellar volume (*p* = .039), corpus callosum cross‐sectional area (*p* = .018), hippocampal volume (*p* = .039), and total surface area (*p* = .008). The correlation with cerebral volume and total surface area was significant after the Benjamini‐Hochberg correction. In contrast with Aβ_1–40_, the sign of each regression coefficient was negative; that is, higher concentrations of p‐tau_181_ tended to be associated with smaller brain sizes.

**Table 2:** Associations of global measures of brain size with CSF biomarkers

	Ab40			Ab42			tau			p‐tau		
	*N*	β	*p*	*N*	β	*p*	*N*	β	*p*	*N*	β	*p*
Cerebral volume	209	0.09	.09	200	−.007	.90	207	.004	.94	204	−.17	.001
Cerebellar volume	209	0.03	.58	200	.002	.98	207	−.03	.53	204	−.11	.039
Corpus callossum cross sectional area	209	0.12	.024	200	−.07	.22	207	.006	.91	204	−.12	.018
Hippocampal volume	209	0.14	.013	200	−.03	.58	207	−.03	.58	204	−.12	.039
Total gray matter thickness	206	0.17	.007	197	.05	.50	204	−.07	.26	201	−.13	.05
Total surface area	206	0.14	.006	197	−.005	.93	204	.005	.93	201	−.14	.008

**Figure 2:**
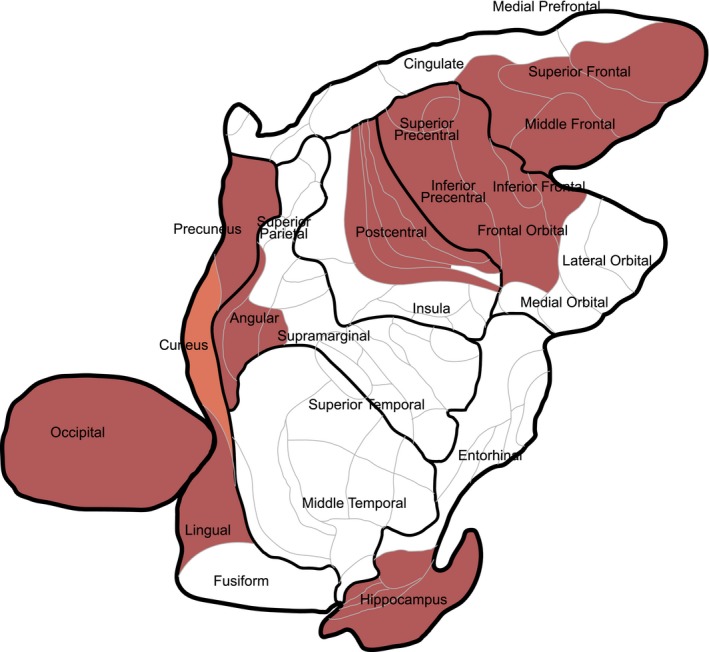
Regional associations of cortical gray matter thickness and CSF Aβ_1–40_ in the vervet brain; coloration of the region indicates suggestive (*p* < .05, orange), and significant association (Benjamini‐Hochberg corrected *p* < .05, red)

Because we observed suggestive association between Aβ_1–40_ and p‐tau_181_ and multiple neuroimaging traits, we further tested for associations between CSF concentrations of Aβ_1–40_ and p‐tau_181_ and regional measures of gray matter thickness and surface area across the cortex (Table S1). MRI measurements were adjusted for age and sex, and the residuals were used for downstream analyses. Hypothesis tests for each association were corrected for multiple comparisons using the method of Benjamini and Hochberg, yielding adjusted p‐values (p_BH_) that control for the false discovery rate. Concentrations of Aβ_1–40_ were correlated with increased gray matter thickness across many brain regions, including the orbital frontal gyri (linear regression coefficient β = 0.26, p_BH_ = 0.002), lingual gyrus (β = 0.19, p_BH_ = 0.040), inferior frontal gyrus (β = 0.17, p_BH_ = 0.040), precuneus (β = 0.18, p_BH_ = 0.040), angular gyrus (β = 0.17, p_BH_ = 0.040), middle frontal gyrus (β = 0.17, p_BH_ = 0.040), inferior precentral gyrus (β = 0.17, p_BH_ = 0.040), superior precentral gyrus (β = 0.16, p_BH_ = 0.044), superior frontal gyrus (β = 0.16, p_BH_ = 0.044), postcentral gyrus (β = 0.16, p_BH_ = 0.044), and lateral occipital gyri (β = 0.16, p_BH_ = 0.047; Table S1, Figure 2). Significant correlations with surface area were limited to the lateral occipital gyrus (β = 0.20, P_BH_ = 0.027). Concentrations of p‐tau_181_ were correlated with decreased gray matter thickness in the fusiform gyrus (β = −0.20, p_BH_ = 0.022), and surface area of the corpus callosum (β = −0.22, p_BH_ = 0.009) and inferior occipital gyri (β = −0.17, p_BH_ = 0.049). The direction of relationship was largely consistent with those in the global brain measures; Aβ_1–40_ was generally correlated with increased gray matter thickness and surface area, while p‐tau_181_ was generally correlated with decreased gray matter thickness and surface area of brain structures. Of these brain regions, only the gray matter thickness in the fusiform gyrus was significantly correlated with age, after correction for multiple comparisons (uncorrected *p* = 3.68 × 10^−14^). Fitting a linear regression model for fusiform gyrus gray matter thickness using both CSF p‐tau_181_ and age at MRI scan as covariates, we found that both age (β = 0.20, *p* = 1.03 × 10^−6^) and p‐tau_181_ (β = 0.20, *p* = .0015) were strong predictors. This pattern remained even after including age at CSF draw (*p* = .00057 for p‐tau_181_), suggesting that the contributions of CSF Aβ_1–40_ and p‐tau_181_ on regional brain volume measurements that we have identified are independent of age.

### 3.4 Heritability and genetic mapping of CSF biomarkers

We first sought to quantify the heritability of CSF Aβ_1–40_, Aβ_1–42_, tau, and p‐tau_181_ concentrations. Narrow‐sense polygenic heritability (*h* ^2^) was estimated using SOLAR, taking into account age and sex as possible covariates. After correcting for relatedness, CSF Aβ_1–40_ (*p* = .001) and p‐tau_181_ (*p* = .01) were significantly correlated with age, but not Aβ_1–42_ (*p* = .46) or tau (*p* = .31). CSF p‐tau_181_ (*p* = .007) but not Aβ_1–40_ (*p* = .32), Aβ_1–42_ (*p* = .25), or tau (*p* = .14) was significantly correlated with sex. The CSF concentration of Aβ_1–40_ was found to be significantly heritable (*h* ^2^ = 0.22, *p* = .01). CSF concentrations of Aβ_1–42_, tau, and p‐tau_181_ were not heritable (*h* ^2 ^= 0.05, *p* = .35; *h* ^2^ = 0.07, *p* = .15; and *h* ^2^ = 0, *p* = .50, respectively).

Because of the demonstrated heritability of CSF Aβ_1–40_ concentration, we then attempted to map genetic loci that could play a role in the phenotypic variation using genome‐wide multipoint linkage analysis. At a LOD score threshold of 3.3, no markers reached genome‐wide significance (Figure S6). Two peaks were suggestive for linkage (LOD > 1.9), including a 9 cM region on vervet chromosome 4, between 67 Mb and 74 Mb (maximum LOD of 2.5) and a 6 cM region on vervet chromosome 12, between 30 Mb and 37 Mb (maximum LOD of 2.2). We additionally performed association as a complementary mapping approach, correcting for family structure using a linear mixed model approach implemented using the EMMAX software (Kang et al., 2010). No markers reached genome‐wide significance, but a suggestive association was identified on chromosome 4 near the proximal end of the linkage peak (minimum *p* = 5.68 × 10^−8^ at marker CAE4_67028748, Figure S7). Genes within 500,000 base pairs of this marker included *MAP1B*,* MRPS27*,* PTCD2*,* ZNF366*,* TNPO1*,* FCHO2*,* TMEM171*, and *TMEM174*.

## 4 DISCUSSION

We report the measurement of CSF biomarkers in a colony of vervet monkeys, a potentially valuable nonhuman primate model for neurodegenerative diseases. This dataset spans vervet monkeys of varying age, from 1 year of age to 27 years of age. For context, vervets typically live to 11–13 years of age in the wild, and about 25 years in captivity (Magden et al., 2015). As AD and other neurodegenerative diseases are highly age‐related in humans, the study of this large dataset is ideal for dissecting the applicability of vervets in human disease.

AD in humans is thought to result in decreased CSF Aβ_1–42_ by accumulation of Aβ_1–42_ in neuritic plaques (Fagan et al., 2006) resulting in reduced drainage through the CSF (Lautner et al., 2014; Strozyk, Blennow, White, & Launer, 2003), and in increased tau by neuronal death (Franz et al., 2003; Itoh et al., 2001) and active secretion (Chai, Dage, & Citron, 2012; Saman et al., 2012). Phospho‐tau, unlike total tau, seems to have greater specificity to neurofibrillary tangle pathology, and does not drastically increase in some neurodegenerative diseases such as Creutzfeldt‐Jakob disease (Itoh et al., 2001) and acute stroke (Hesse et al., 2001). The relationship of CSF Aβ_1–40_ with AD has been less clear, though CSF Aβ_1–40_ has been reported as decreased in CAA (Verbeek et al., 2009). Here, we found that reductions in global measures of brain size (such as cerebral volume and total brain surface area) were associated with reduced CSF Aβ_1–40_ and elevated CSF p‐tau_181_. Taken together with previous observations of age‐related amyloid plaques in the vervet (Kalinin et al., 2013; Lemere et al., 2004), the relationships with Aβ_1–40_ and p‐tau_181_ are consistent with a scenario of age‐related accumulation of amyloid plaques (in parenchyma and/or vasculature) corresponding to cerebral atrophy in the vervet. Though previous studies have found limited phospho‐tau immunoreactivity and few neurofibrillary tangles in the vervet brain (Lemere et al., 2004), the fact that elevations in p‐tau_181_ levels in CSF are also correlated with changes in brain morphometry suggest that tau pathology may also play a role.

These findings are reminiscent of the observed relationship between CSF biomarkers and neuroimaging measurements in aging and neurodegenerative disease in humans. Previous work has demonstrated associations between longitudinal changes in Aβ_1–42_ and p‐tau_231_ and hippocampal volume in AD and mild cognitive impairment (Chou et al., 2009; De Leon et al., 2006; Hampel et al., 2005; Schuff et al., 2009) as well as in healthy brain aging (Fjell et al., 2010) in humans. However, we identified significant associations of Aβ_1–40_, not Aβ_1–42_, with age, gray matter thickness, and other measures of brain size. In vervets (Lemere et al., 2004) and in humans (Attems, Jellinger, Thal, & Van Nostrand, 2011; Attems, Lintner, & Jellinger, 2004; Gravina et al., 1995), Aβ_1–40_ predominates in CAA while Aβ_1–42_ predominates in parenchymal plaques, and this neuropathology is reflected in differences in the concentrations of these Aβ species in CSF. Furthermore, the brain regions most associated with decreased Aβ_1–40_ in this study in vervet (predominant occipital cortical involvement, as well as precuneus and frontal cortex) mirror the distribution of CAA and subsequent neurodegeneration in humans (Attems et al., 2011; Vinters & Gilbert, 1983). Because of these converging lines of evidence, we speculate that the changes in Aβ_1–40_ observed correlate with CAA pathology, although further study is required to confirm this hypothesis. In a subset of this cohort containing 22 vervets ranging from 11 to 26 years of age with neuropathology, amyloid plaque burden increased with age and correlated inversely with CSF Aβ_1–42_ levels (Dr. Suzanne Craft, *personal communication*). The lack of association with CSF Aβ_1–42_ with age and with brain regions may reflect inconsistent amyloid plaque burden in the aged vervet, compared to CAA pathology.

Moreover, we find evidence for the heritability of CSF Aβ_1–40_ concentrations in the vervet. Linkage analysis identified two suggestive linkage peaks: a wide 9 cM peak on chromosome 4, and a narrow 6 cM peak on chromosome 12. The lack of statistical significance in linkage analysis despite the moderate heritability of CSF Aβ_1–40_ concentration may suggest a highly polygenic contribution to the phenotype. While the signal did not reach genome‐wide significance and spanned a wide genomic range, precluding identification of a single causal gene or variant, our work provides a basis for further fine mapping of these regions. Potential candidate genes in the region include *PTCD2* and *MAP1B*, at the proximal end of the linkage peak and approximately 330 and 470 kilobases from the top associated marker. The pentatricopeptide repeat domain 2 gene, encoded by *PTCD2*, has been observed to be accumulated in AD brain in citrullinated form, and autoantibodies against *PTCD2* have been observed in AD patient serum (Acharya et al., 2012). The microtubule‐associated protein 1B, encoded by *MAP1B*, has been described to bind tau and Aβ (Gevorkian et al., 2008; Hasegawa, Arai, & Ihara, 1990). In humans, genome‐wide association studies have demonstrated genetic loci that affect CSF biomarkers, the most robust being the region surrounding the *APOE* gene (Cruchaga et al., 2013). The linkage analysis here may provide a foothold to discover analogous loci for vervets, but further work remains to validate and pinpoint them.

One observed anomaly was the fact that concentration of total tau was lower than that for p‐tau_181_ in many CSF specimens. This was unexpected because the phosphorylated species is also reactive to the antibodies against total tau, and therefore the concentration of total tau should be an upper bound for that of the phosphorylated species. Because the calibration standards and controls were derived from human tau, we postulated that non‐homology in the epitope region may confound the assay. Indeed, we found an amino acid difference in the vervet tau ortholog in the AT120 epitope region that may affect the specificity of the antibody. We speculate that this lack of homology accounts for the aberrant total tau levels that were observed and, therefore, the measurement of the total tau using the AlzBio3 assay in vervets must be interpreted with caution. The epitope regions for all of the other antibodies used to quantify levels of Aβ_1–40_, Aβ_1–42_, and p‐tau_181_ were identical between human and vervet.

Another limitation of the study is the fact that neuroimages and CSF samples were not acquired simultaneously; the interval between imaging and CSF collection was variable, but imaging was performed approximately 5 years before CSF collection for each sample. Somewhat mitigating the possibility for extensive confounding, we had previously demonstrated high heritability of neuroimaging phenotypes, in some instances approaching 100%; therefore, environmental influences are likely to be low (Fears et al., 2009). We were able to identify associations between CSF markers and neuroimaging despite this gap, which could introduce further variability into the analysis; therefore, the associations we positively identified are likely to reflect biology. However, the temporal relationship and relative effect sizes of the CSF marker‐neuroimaging relationship are not known. In other words, differences in CSF Aβ and tau concentrations on a population level in vervet can be confidently correlated to aging and neurodegeneration (after controlling for aging), but the sensitivity and degree of correlation remains unclear. Although we cannot make a conclusion about the prognostic power of CSF markers, preliminary data suggests that CSF Aβ_1–42_ is a predictor of parenchymal amyloid plaques (Suzanne Craft, *personal communication*). Interestingly, neither cerebral volume or p‐tau_181_ were associated with age across the wide range of ages in the vervet cohort; however, the strongest association was detected between p‐tau_181_ and cerebral volume, suggesting that at least some of the associations were not driven by dependency on age. Furthermore, frank cognitive deficits were not observed or ascertained in the vervet subjects, and the study design was cross‐sectional; correlation with measures of brain structure and function in future longitudinal studies may provide additional insight into the extent of neurodegeneration.

In vervets, CSF Aβ_1–40_ decreases with advancing age, correlates with global and regional measures of brain size, and has a large heritable component, suggesting its utility as a biomarker of AD‐ or CAA‐like changes in this potentially valuable animal model. Aβ_1–40_ was most closely associated with decreases in gray matter thickness in the frontal lobe and hippocampus, mirroring the regional vulnerability patterns of AD. However, structural associations were also observed in the occipital lobe and precuneus, regions that are generally spared early in Alzheimer's disease and more reminiscent of the distribution of pathology in CAA. Age‐related cerebral atrophy in the vervet likely involves β‐amyloid pathological processes, and Aβ_1–40_ and p‐tau_181_ appear to be robust biomarkers of these processes, though their sensitivities and correlations to disease pathology remain important open questions. We have also mapped suggestive linkage peaks on vervet chromosomes 4 and 12 that correspond to CSF Aβ_1–40_ concentration and may represent a modifier of neurodegeneration. The aged vervet therefore has the potential to advance our understanding of the genetics, pathogenesis, and treatment of neurodegenerative disease.

## CONFLICT OF INTEREST

None declared.
