# Supplementary material for: Neurodegenerative disease biomarkers Aβ1–40, Aβ1–42, tau, and p‐tau181 in the vervet monkey cerebrospinal fluid: Relation to normal aging, genetic influences, and cerebral amyloid angiopathy
Source: Brain Behav. 2018 Jan 13;8(2):e00903. doi: 10.1002/brb3.903 (PMC5822592; doi:10.1002/brb3.903)
Supplement: Supplementary file 1 [file BRB3-8-e00903-s001.docx]

Supplementary Figure 1. Distribution of the concentrations of four CSF biomarkers in the vervet cohort, a) Aβ_1-40_, b) Aβ_1-42_, c) tau, and d) p-tau_181_.

Supplementary Figure 2. Correlation between the age at CSF collection and four CSF biomarkers: a) Aβ_1-40_, b) Aβ_1-42_, c) tau, and d) p-tau_181_. CSF biomarker concentrations are given in pg/ml.


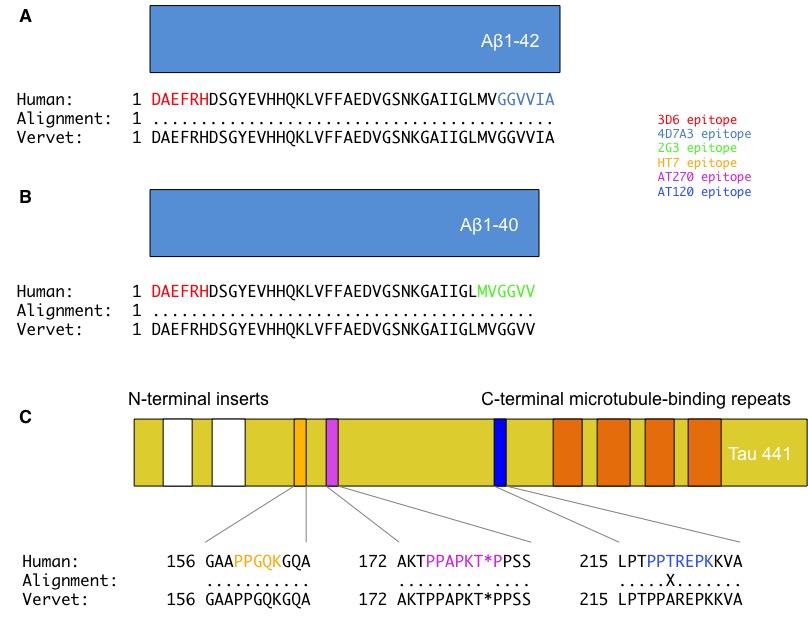


Supplementary Figure 3. Overlap of antibody epitope regions and vervet species-specific genetic differences and vervet polymorphisms in a) Aβ_1-42_, b) Aβ_1-40_, and c) tau.

Supplementary Figure 4. Correlation between CSF biomarker measurements and vervet coding region polymorphisms, APP Ala15Ser, MAPT Gly148Arg, and MAPT Leu213Pro.

Supplementary Figure 5. Age distribution of vervets in the study. The ages were included from both the time at CSF draw (blue) and the time at MRI scan (yellow). A "common cohort" included animals with data from both CSF draw and MRI scan (dark), and study-specific cohorts included animals with either CSF draw or MRI scan, but not both (light).

Supplementary Figure 6. LOD scores from linkage mapping of Aβ_1-40_. LOD > 3.3 was considered significant (no markers met this threshold); LOD > 1.9 was considered suggestive (red dashed line; linkage peaks on chromosomes 4 and 12 met this threshold).


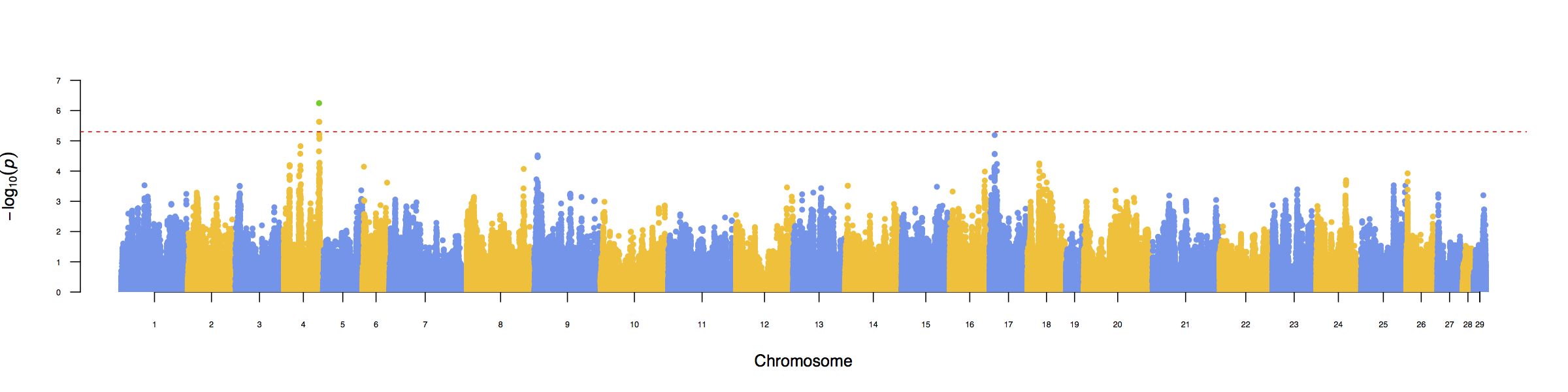


Supplementary Figure 7. P-values from genome-wide association of Aβ_1-40_. A p-value threshold < 5x10^-8^ was considered genome-wide significant (no loci met this threshold); p < 5x10^-6^ was considered suggestive for association (red line; a locus on chromosome 4 met this threshold).

|  |  | **Aβ40** | | | **p-tau_181_** | | |
| --- | --- | --- | --- | --- | --- | --- | --- |
|  |  | **beta** | **p** | **FDR** | **beta** | **p** | **FDR** |
| **Angular** | GT | 0.17 | 0.008 | 0.04 | -0.081 | 0.23 | 0.36 |
|  | SA | 0.13 | 0.031 | 0.11 | -0.05 | 0.43 | 0.52 |
| **Ant_Cingulate** | GT | 0.069 | 0.27 | 0.34 | -0.1 | 0.12 | 0.34 |
|  | SA | 0.081 | 0.21 | 0.3 | -0.0029 | 0.96 | 0.98 |
| **Corpus_Callosum** | GT | -0.02 | 0.76 | 0.76 | -0.074 | 0.27 | 0.36 |
|  | SA | 0.1 | 0.1 | 0.21 | -0.22 | 0.00031 | 0.0091 |
| **Cuneus** | GT | 0.14 | 0.037 | 0.089 | -0.13 | 0.056 | 0.34 |
|  | SA | 0.088 | 0.16 | 0.28 | -0.088 | 0.18 | 0.3 |
| **Entorhinal** | GT | 0.069 | 0.31 | 0.36 | -0.024 | 0.73 | 0.76 |
|  | SA | 0.11 | 0.069 | 0.18 | -0.033 | 0.58 | 0.65 |
| **Frontal_Orbital** | GT | 0.26 | 6.9x10^-5^ | 0.002 | -0.062 | 0.36 | 0.42 |
|  | SA | 0.063 | 0.23 | 0.31 | -0.08 | 0.13 | 0.24 |
| **Fusiform** | GT | 0.046 | 0.44 | 0.47 | -0.2 | 0.00077 | 0.022 |
|  | SA | 0.094 | 0.11 | 0.21 | -0.1 | 0.09 | 0.22 |
| **Gyrus_Rectus** | GT | 0.078 | 0.27 | 0.34 | -0.08 | 0.25 | 0.36 |
|  | SA | 0.073 | 0.24 | 0.31 | -0.063 | 0.29 | 0.42 |
| **Inf_Frontal** | GT | 0.17 | 0.0069 | 0.04 | -0.087 | 0.19 | 0.36 |
|  | SA | 0.072 | 0.17 | 0.28 | -0.051 | 0.32 | 0.43 |
| **Inf_Occipital** | GT | 0.13 | 0.044 | 0.098 | -0.13 | 0.062 | 0.34 |
|  | SA | 0.14 | 0.013 | 0.069 | -0.17 | 0.0034 | 0.049 |
| **Inf_Precentral** | GT | 0.16 | 0.0097 | 0.04 | -0.091 | 0.14 | 0.36 |
|  | SA | 0.11 | 0.049 | 0.14 | -0.11 | 0.056 | 0.16 |
| **Inf_Temporal** | GT | 0.06 | 0.38 | 0.42 | -0.077 | 0.27 | 0.36 |
|  | SA | 0.079 | 0.17 | 0.28 | -0.12 | 0.029 | 0.14 |
| **Insula** | GT | 0.093 | 0.12 | 0.21 | -0.09 | 0.16 | 0.36 |
|  | SA | 0.05 | 0.42 | 0.45 | -0.032 | 0.61 | 0.65 |
| **Lat_Orbital** | GT | 0.083 | 0.17 | 0.26 | -0.0035 | 0.95 | 0.95 |
|  | SA | 0.099 | 0.1 | 0.21 | -0.0016 | 0.98 | 0.98 |
| **Lingual** | GT | 0.19 | 0.0032 | 0.04 | -0.073 | 0.26 | 0.36 |
|  | SA | 0.17 | 0.0088 | 0.069 | -0.13 | 0.04 | 0.16 |
| **Med_Orbital** | GT | 0.091 | 0.17 | 0.26 | -0.068 | 0.3 | 0.37 |
|  | SA | 0.14 | 0.014 | 0.069 | 0.035 | 0.56 | 0.65 |
| **Mid_Frontal** | GT | 0.17 | 0.0095 | 0.04 | -0.07 | 0.3 | 0.37 |
|  | SA | 0.056 | 0.26 | 0.32 | -0.11 | 0.028 | 0.14 |
| **Mid_Temporal** | GT | 0.09 | 0.19 | 0.26 | -0.13 | 0.06 | 0.34 |
|  | SA | 0.11 | 0.039 | 0.13 | -0.11 | 0.046 | 0.16 |
| **Occipital** | GT | 0.16 | 0.018 | 0.047 | -0.14 | 0.038 | 0.34 |
|  | SA | 0.2 | 0.00091 | 0.027 | -0.059 | 0.35 | 0.44 |
| **Post_Cingulate** | GT | 0.096 | 0.12 | 0.21 | -0.088 | 0.16 | 0.36 |
|  | SA | 0.054 | 0.35 | 0.39 | -0.13 | 0.019 | 0.14 |
| **Post_Parahippo** | GT | 0.07 | 0.3 | 0.36 | -0.11 | 0.11 | 0.34 |
|  | SA | 0.0067 | 0.91 | 0.91 | -0.075 | 0.23 | 0.36 |
| **Postcentral** | GT | 0.16 | 0.015 | 0.044 | -0.11 | 0.087 | 0.34 |
|  | SA | 0.093 | 0.095 | 0.21 | -0.088 | 0.11 | 0.22 |
| **Precuneus** | GT | 0.18 | 0.0069 | 0.04 | -0.087 | 0.19 | 0.36 |
|  | SA | 0.062 | 0.31 | 0.36 | -0.065 | 0.29 | 0.42 |
| **Prepyriform** | GT | 0.047 | 0.49 | 0.5 | -0.062 | 0.38 | 0.42 |
|  | SA | 0.038 | 0.55 | 0.57 | -0.067 | 0.3 | 0.42 |
| **Sup_Frontal** | GT | 0.16 | 0.014 | 0.044 | -0.084 | 0.2 | 0.36 |
|  | SA | 0.065 | 0.24 | 0.31 | -0.089 | 0.098 | 0.22 |
| **Sup_Parietal** | GT | 0.12 | 0.079 | 0.16 | -0.059 | 0.39 | 0.42 |
|  | SA | 0.13 | 0.024 | 0.1 | -0.11 | 0.071 | 0.19 |
| **Sup_Precentral** | GT | 0.16 | 0.013 | 0.044 | -0.074 | 0.26 | 0.36 |
|  | SA | 0.14 | 0.014 | 0.069 | -0.13 | 0.029 | 0.14 |
| **Sup_Temporal** | GT | 0.087 | 0.18 | 0.26 | -0.12 | 0.071 | 0.34 |
|  | SA | 0.065 | 0.19 | 0.29 | -0.095 | 0.054 | 0.16 |
| **Supramarginal** | GT | 0.11 | 0.1 | 0.2 | -0.11 | 0.1 | 0.34 |
|  | SA | 0.14 | 0.011 | 0.069 | -0.088 | 0.13 | 0.24 |

Supplementary Table 1. Associations of gray matter thickness and surface area of cortical regions with CSF biomarkers.
